# Supplementary material for: Developmental regulation of canonical and small ORF translation from mRNAs
Source: Genome Biol. 2020 May 29;21:128. doi: 10.1186/s13059-020-02011-5 (PMC7260771; doi:10.1186/s13059-020-02011-5)
Supplement: Supplementary file 1 — Additional file 1. Patraquim_Supplemental_Information_Additional File 1.pdf: Supplementary Figures and Tables. [file 13059_2020_2011_MOESM1_ESM.pdf]

Additional File 1: Figure S1

A

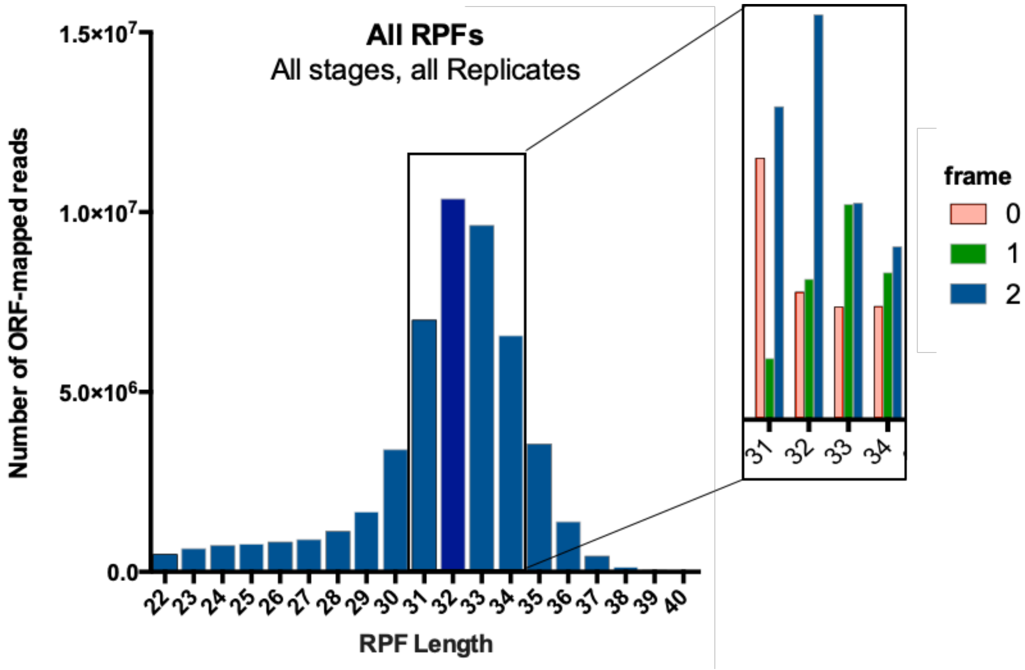

B

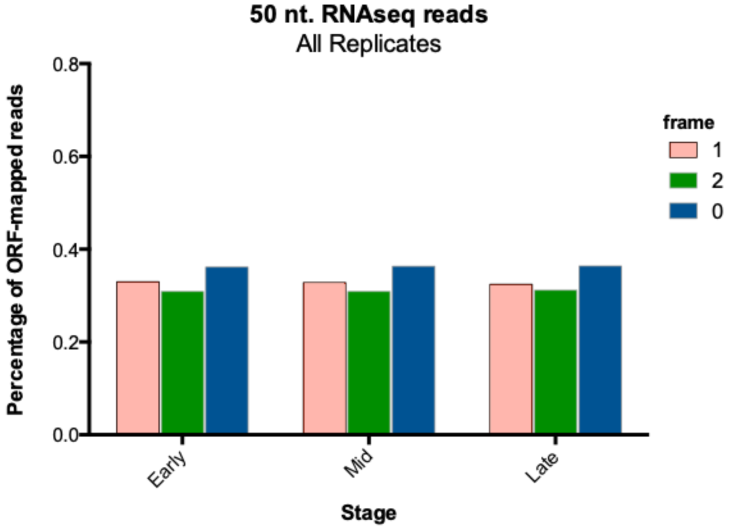

Additional File 1: Figure S2

A

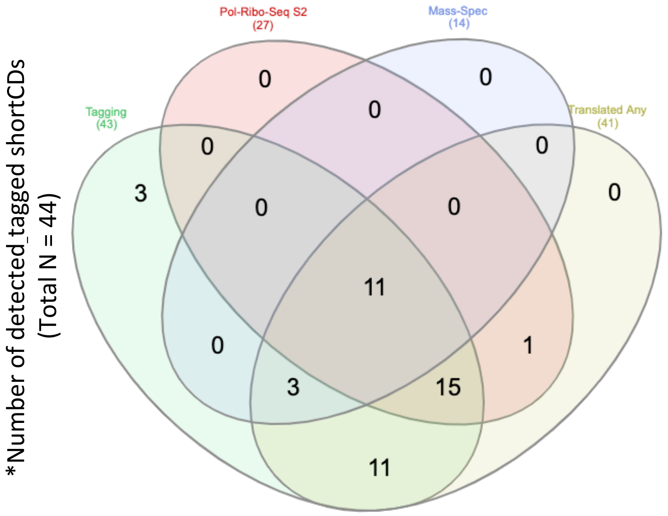

B

244 genes (529 ORFs) – Up Early-to-Mid

Terms	p-Value	Matches
ventral nerve cord	8.222461e-4	52
embryonic dorsal epidermis	8.762168e-4	36
embryonic epipharynx	0.002898	21
embryonic hypopharynx	0.006638	21
embryonic brain	0.007916	49
embryonic foregut	0.015561	23
embryonic ventral epidermis	0.032294	30
embryonic esophagus	0.037462	14

C

122 genes (136 ORFs)- Up Maternal-to-Early

Terms	p-Value	Matches
ubiquitous	1.125900e-5	34
maternal	4.074426e-4	47

D

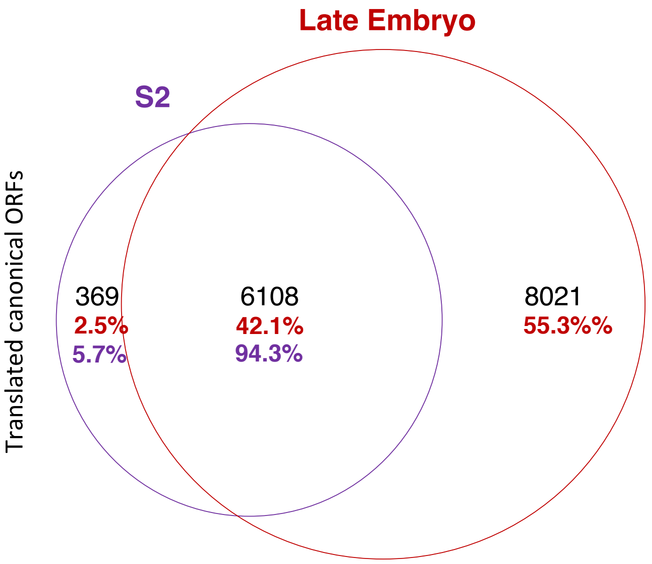

Additional File 1: Figure S3

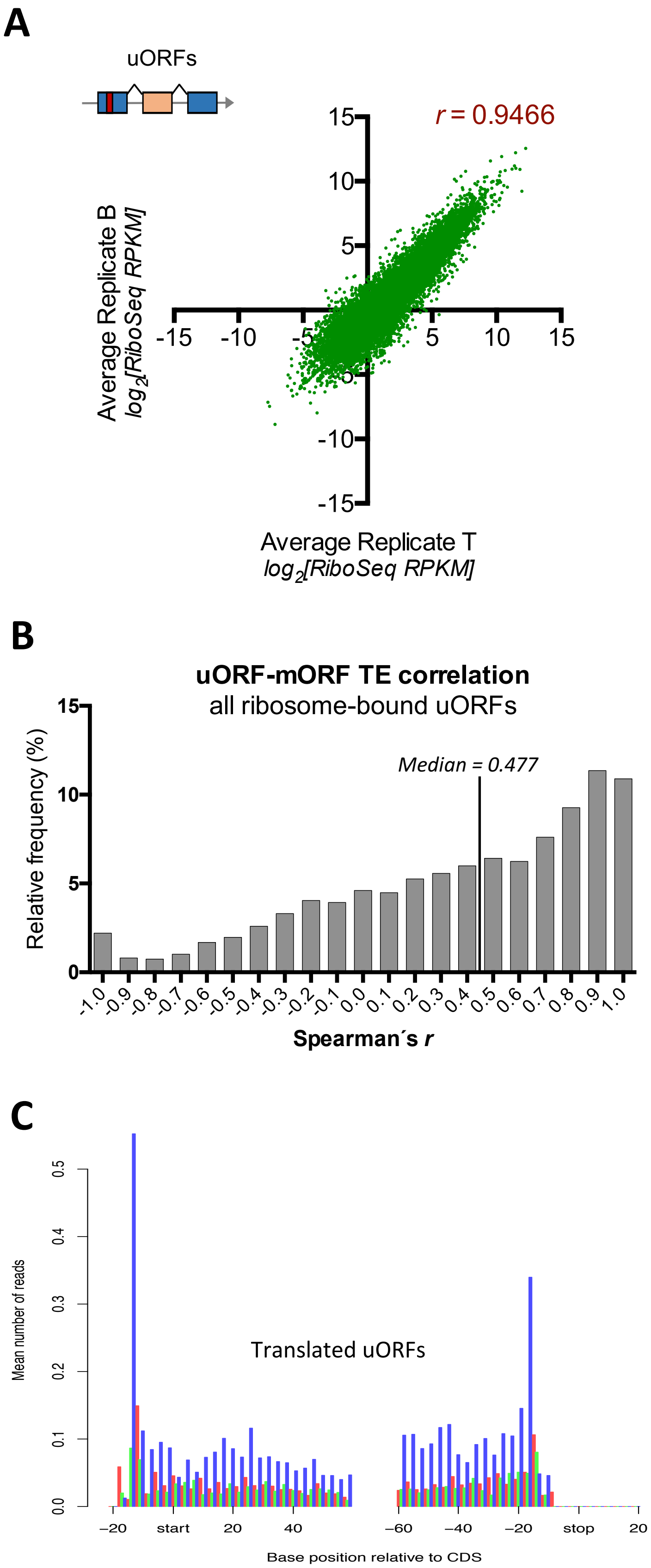

Additional File 1: Table S1

Sequencing	Replica	Stage	*Reads*		
			**Raw reads**	**norRNA (%)**	**Genome-mapped (%)**
RiboSeq	T	Early	285.529.728	14.8	73
	B	Early	322.959.034	41.6	74.5
	T	Mid	178.496.111	41.2	75.2
	B	Mid	170.312.394	29.3	82.1
	T	Late	110.156.905	72.4	77.2
	B	Late	158.351.452	47.2	75
RNA-Seq	T	Early	39.817.649	51.8	74.8
	B	Early	99.986.496	87.6	84.5
	T	Mid	54.333.403	44.5	56
	B	Mid	170.312.394	49.8	79.8
	T	Late	68.857.596	53.5	53.9
	B	Late	66.245.016	74.7	71.9

Additional File 1: Table S2

	Early_RNA-Seq_Flybase	Mid_RNA-Seq_Flybase	Late_RNA-Seq_Flybase	Early_Riboseq_T	Early_Riboseq_B	Mid_Riboseq_T	Mid_Riboseq_B	Late_Riboseq_T	Late_Riboseq_B	Early_RNA-Seq_T	Early_RNA-Seq_B	Mid_RNA-Seq_T	Mid_RNA-Seq_B	Late_RNA-Seq_T	Late_RNA-Seq_B	S2_Riboseq	S2_RNA-Seq	Unfertilized_Egg_Riboseq	Unfertilized_Egg_RNA-Seq
Early_RNA-Seq_Flybase	0	0.87	0.64	0.93	0.92	0.68	0.71	0.53	0.53	0.83	0.94	0.66	0.69	0.54	0.62	0.81	0.8	0.86	0.86
Mid_RNA-Seq_Flybase	0.87	0	0.82	0.84	0.85	0.86	0.87	0.68	0.67	0.76	0.84	0.82	0.84	0.7	0.71	0.76	0.74	0.75	0.72
Late_RNA-Seq_Flybase	0.64	0.82	0	0.65	0.67	0.87	0.82	0.89	0.86	0.65	0.65	0.82	0.83	0.85	0.83	0.61	0.58	0.57	0.54
Early_Riboseq_T	0.93	0.84	0.65	0	0.99	0.75	0.79	0.61	0.63	0.85	0.95	0.72	0.73	0.58	0.69	0.8	0.78	0.88	0.83
Early_Riboseq_B	0.92	0.85	0.67	0.99	0	0.78	0.82	0.64	0.66	0.86	0.95	0.74	0.76	0.61	0.72	0.79	0.77	0.86	0.81
Mid_Riboseq_T	0.68	0.86	0.87	0.75	0.78	0	0.98	0.88	0.87	0.71	0.73	0.91	0.9	0.82	0.83	0.66	0.63	0.63	0.59
Mid_Riboseq_B	0.71	0.87	0.82	0.79	0.82	0.98	0	0.84	0.85	0.74	0.76	0.9	0.91	0.79	0.82	0.69	0.66	0.67	0.62
Late_Riboseq_T	0.53	0.68	0.89	0.61	0.64	0.88	0.84	0	0.99	0.64	0.61	0.82	0.82	0.88	0.89	0.57	0.54	0.52	0.48
Late_Riboseq_B	0.53	0.67	0.86	0.63	0.66	0.87	0.85	0.99		0.65	0.62	0.81	0.81	0.86	0.89	0.58	0.55	0.54	0.5
Early_RNA-Seq_T	0.83	0.76	0.65	0.85	0.86	0.71	0.74	0.64	0.65	0	0.92	0.83	0.83	0.72	0.82	0.74	0.75	0.76	0.79
Early_RNA-Seq_B	0.94	0.84	0.65	0.95	0.95	0.73	0.76	0.61	0.62	0.92	0	0.75	0.79	0.64	0.73	0.82	0.81	0.87	0.88
Mid_RNA-Seq_T	0.66	0.82	0.82	0.72	0.74	0.91	0.9	0.82	0.81	0.83	0.75	0	0.95	0.88	0.91	0.64	0.63	0.61	0.6
Mid_RNA-Seq_B	0.69	0.84	0.83	0.73	0.76	0.9	0.91	0.82	0.81	0.83	0.79	0.95	0	0.89	0.89	0.68	0.68	0.63	0.62
Late_RNA-Seq_T	0.54	0.7	0.85	0.58	0.61	0.82	0.79	0.88	0.86	0.72	0.64	0.88	0.89	0	0.91	0.57	0.56	0.5	0.49
Late_RNA-Seq_B	0.62	0.71	0.83	0.69	0.72	0.83	0.82	0.89	0.89	0.82	0.73	0.91	0.89	0.91	0	0.61	0.6	0.59	0.57
S2_Riboseq	0.81	0.76	0.61	0.8	0.79	0.66	0.69	0.57	0.58	0.74	0.82	0.64	0.68	0.57	0.61	0	0.96	0.82	0.83
S2_RNA-Seq	0.8	0.74	0.58	0.78	0.77	0.63	0.66	0.54	0.55	0.75	0.81	0.63	0.68	0.56	0.6	0.96	0	0.81	0.82
Unfertilized_Egg_Riboseq	0.86	0.75	0.57	0.88	0.86	0.63	0.67	0.52	0.54	0.76	0.87	0.61	0.63	0.5	0.59	0.82	0.81	0	0.93
Unfertilized_Egg_RNA-Seq	0.86	0.72	0.54	0.83	0.81	0.59	0.62	0.48	0.5	0.79	0.88	0.6	0.62	0.49	0.57	0.83	0.82	0.93	0

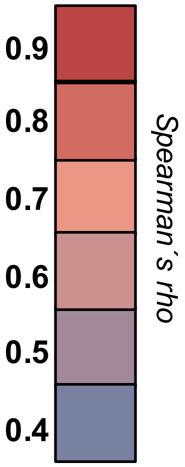

Additional File 1: Table S3

A

Measure	Stage	Replica	*tal-1A*	*tal-2A*	*tal-3A*	*tal-AA*
tal			*ORF*			
RNA-Seq RPKM	Early	T	19.75	87.08	154.42	45.70
	Early	B	10.28	102.47	139.96	61.70
	Mid	T	110.55	600.49	1560.27	130.65
	Mid	B	57.59	792.04	508.15	335.57
	Late	T	4.06	36.12	29.80	12.15
	Late	B	1.49	24.80	40.17	9.74
Ribo-Seq RPKM	Early	T	3.60	7.20	7.20	8.51
	Early	B	19.53	35.30	68.72	33.46
	Mid	T	36.94	34.89	59.52	47.02
	Mid	B	76.78	147.40	293.56	144.52
	Late	T	4.19	4.19	12.58	9.65
	Late	B	13.27	39.81	53.07	31.50
TE	Early	T	5.48	12.09	21.43	5.37
	Early	B	0.53	2.90	2.04	1.84
	Mid	T	2.99	17.21	26.21	2.78
	Mid	B	0.75	5.37	1.73	2.32
	Late	T	0.97	8.62	2.37	1.26
	Late	B	0.11	0.62	0.76	0.31
translation probability (binomial p)	**Early**	Both	**4.07E-05**	**1.67E-09**	**9.67E-08**	**5.26E-07**
	**Mid**	Both	**8.02E-06**	**1.33E-14**	**4.48E-13**	**7.22E-07**
	**Late**	Both	7.04E-01	1.27E-01	**3.60E-05**	7.39E-01
translational regulation (Z-ratio)	**Early-Mid**	Both	1.22E-01	3.26E+00	2.93E+00	2.05E-01
	**Mid-Late**	Both	-5.01E-01	-3.98E+00	-4.36E+00	-1.05E+00
Conservation (phyloP)			2.5	3.1	2.8	2

B

Measure	Stage	Replica	*scl-A*	*sclB*
scl			*ORF*	
RNA-Seq RPKM	Early	T	0.00	1.44
	Early	B	1.24	0.75
	Mid	T	5.94	17.24
	Mid	B	6.63	20.20
	Late	T	6.36	3.35
	Late	B	9.69	20.92
Ribo-Seq RPKM	Early	T	0.00	1.08
	Early	B	0.57	0.56
	Mid	T	5.20	50.05
	Mid	B	2.24	11.92
	Late	T	74.74	140.88
	Late	B	55.62	59.71
TE	Early	T	N/A	0.75
	Early	B	0.46	0.74
	Mid	T	0.87	2.90
	Mid	B	0.34	0.59
	Late	T	11.75	42.01
	Late	B	5.74	2.85
translation probability (binomial p)	**Early**	Both	3.33E-01	1.00E+00
	**Mid**	Both	**8.28E-03**	9.07E-02
	**Late**	Both	**1.03E-43**	**5.32E-06**
translational regulation (Z-ratio)	**Early-Mid**	Both	1.70E-01	2.70E-01
	**Mid-Late**	Both	2.03E+00	3.30E+00
Conservation (phyloP)			2.6	2.9

Additional File 1: Table S4

ORF class		**Transcribed** ORFs		Total
**Canonical**	14469	16273	16332	17280
**shortCDS**	402	436	471	490
**uORF**	8454	9858	9069	11710
		**Ribo-Bound** ORFs		
**Canonical**	12732	15650	15544	16858
**shortCDS**	295	386	405	443
**uORF**	5691	6669	5416	8346
		**Translated** ORFs		
**Canonical**	12037	13796	14129	15738
**shortCDS**	248	249	293	343
**uORF**	909	533	523	1258
	Early	*Mid*	*Late*	*Embryo*

Additional File 1: Sup. Figure Legends

Figure S1 – Poly-RiboSeq read length distribution and transcriptome-wide framing. A Distribution of Poly-Ribo-Seq genome-mapped ribosome-protected fragment (RPFs) lengths; codon-framing of predominant lengths shown in inset. **B** negative control: of codon-framing in genome-mapped RNA-Seq reads fails to show dominance in any of the frames.

Figure S2 –A Detectability of mRNA translation across techniques. Number of sCDSs detected by Mass-spectrometry, Poly-Ribo-Seq and FLAG-Tagging (Total N=44). **B-C** Significantly-enriched gene expression patterns in the translationally-upregulated canonical genes the Early-to-Mid and Maternal-to-Early developmental transitions respectively (*p-value* of BGDP expression annotations after Benjamini-Hochberg procedure). **D** S2-cell canonical translation. Comparison of translated canonical ORFs in S2 cells and the late embryo stages from which this cell culture originated [28].

Figure S3 – uORF A Comparison of uORF translation from T and B replicas (in $\log_2$RPKM) show a very high correlation (Spearman's *r*=0.9466). **B** Histogram of individual Spearman's correlation *r* between TE of ribosome-bound uORFs and their corresponding mORF across embryogenesis. The overall positive correlation has a median of *r*=0.477 (binning=0.1 windows). **C**. Metagene plot for codon framing of 32-nt. ribosome footprints across all translated uORFs detected by the binomial test in this study, showing three-nucleotide periodicity (framing) in the third position of each codon (frame 2, blue) in a pattern indistinguishable from annotated canonical ORFs (see Figure 2B).

Additional File 1: Sup. Table Legends

Table S1 – Sequencing statistics for embryonic Poly-RiboSeq and RNA-Seq datasets reported in this study. Number of raw reads per replica per stage and percentage of remaining reads after ribosomal RNA subtraction ("norRNA") and genome-mapping (see methods).

Table S2 – Heatmap of correlations across RNA-Seq and RiboSeq samples and replicates Spearman correlation values (*r*) for canonical ORF RPKMs across all RNA-Seq and RiboSeq replicates analysed in this study. All correlations were significant ($p<0.0001$). T and B denote the two biological replicates used in this study. Early, Mid and Late denote Embryonic windows 0-8h, 8-16h and 16-24h, respectively. "Flybase" RNA-Seq refers to modENCODE data [40]. S2 refers to S2-cell datasets (see methods). Unfertilized Egg corresponds to previously-published data [18].

Tables S3 - Gene expression, regulation and conservation statistics for distinct small ORFs in polycistronic mRNAs. A The *tal* polycistronic mRNA shows quantitative differences in translation efficiency, probability and regulation, as well as conservation, across its four translated small ORFs in correlation with its function in trachea and epidermis development during mid-embryogenesis [30]. **B** The *scl* locus contains two cistronic small ORFs that show similar but quantitatively different levels of translation in correlation with its function in late embryogenesis [39]. Numbers shown in red pass each relevant filter in our pipeline.

Table S4 – Number of Transcribed, Ribosome-Bound and Translated ORFs by Class and embryonic stage. Different ORF classes show distinct amounts of transcription, ribosome binding and translation across stages.
