# Supplementary material for: Developmental regulation of canonical and small ORF translation from mRNAs
Source: Genome Biol. 2020 May 29;21:128. doi: 10.1186/s13059-020-02011-5 (PMC7260771; doi:10.1186/s13059-020-02011-5)
Supplement: Supplementary file 5 — Additional file 5. Review history. [file 13059_2020_2011_MOESM5_ESM.docx]

**First Round of Review**

**Reviewer 1**

The manuscript describdes an improved poly-ribo-seq workflow (improving on their own work) to identify smORFs during embryogenesis. Key findings include the discovery of thousands of non-annotated ORFs, and many mRNAs that are regulated during emboryogensis. Furthermore, they find sites of non-productive (i.e. non translated) ribosome binding sites, mostly in 5-UTRs which indicate an unprecedented scale of post-transcriptional translation regulation. The data quality in the manuscript is excellent and the claims are all supported by the data. The take home message that uORFs are important regulators and buffers of gene expression, and that this type of regulation is important in embryogenesis is powerful and important. It would have been interesting if they could have used some of the more common methods for Ribo-Seq analysis such as ribotaper or riborf, but these would not change the conclusions of the paper and therefore are not necessary for this manuscript.

**Reviewer 2**

This article examines translation patterns during Drosophila embryogenesis using ribosome profiling data. The authors use a variation of the technique in which they first enrich for polysomes and then perform RNAse treatment and recovery of ribosome-protected RNA fragments (Poly-Ribo-Seq). They analyse three different time points and compare the data to S2 cell lines and available proteomics data. The results are reported separately for annotated canonical ORFs, annotated small ORFs and upstream ORFs. The study provides a global view of translation in Drosophila embryos and investigates the possible regulation of translation by uORFs. The results are interesting and provide new data to understand translation regulation in Drosophila. The data support that, in general, uORF translation does not have a repressive effect on the translation of the main ORF. The authors conclude that uORFs may have a buffering effect on the translation of the main ORF and that they could represent evolutionary intermediates of fully-fledged coding sequences.

Major comments:

1. The authors use the binomial distribution to determine if an ORF shows an excess of in-frame reads when compared to the expectation (1/3 of reads in each frame). An important consideration is that this test (as many others) is going to be less sensitive for small ORFs than for longer ORFs and this could have a strong impact on the interpretation of the results, especially with regards to uORFs. Short ORFs are going to have less mapped Ribo-Seq reads than longer ORFs (for the same RPKM). But the longer the sequence the higher the power of the test because the number of reads is going to be larger for longer ORFs. For example if we have the following distribution in the three frames - 8,2,2 - and this is compared to the random one - 4,4,4 - the test is going to be less significant than if we compare 80,20,20 to 40,40,40 (in a longer sequence with the same read coverage). One of the main conclusions of the study is that most uORF-ribosome associations are not productive (they are not consistent with translation) but before reaching such conclusion one should assess if this could be caused, at least partly, to the above mentioned effect.

2. It is not clear how uORFs are defined. For example, what happens when there are several overlapping uORFs? In the case of annotated ORFs alternative overlapping ORF (say in another frame) are automatically discarded, as only the annotated ORF is considered. It should be explained clearly how uORFs are defined on the sequences and how this is different (if it is) from how ORFs are defined in other mRNA regions.

3. The abstract states: "Finally, we show that the main function of upstream short ORFs is to buffer the translation of canonical ORFs". This seems an overstatement as it is based on a single observation - genes with no uORF have more TE variation in the main ORF - which could be due to other causes. I suggest replacing that sentence for something like "the results are consistent with..".

4. The concept of "canonical translation" should be defined, I think the authors mean annotated coding sequences, this should be clarified.

Minor comments:

The text is difficult to read in some places. For example:

Line 134: FPs is used for the first time here here as an acronym, write the whole word/sentence.

Line 135: "we obtained higher yield". Higher than what?

Line 181-183: Could you explain the example of tarsal-less in more detail?

Line 190: "to indicate signal above error and background". I do not think that high correlation across replicates indicates this. It indicates reproducibility.

Line 223: "and because only 32 nt FP were used here". I fail to see the relationship between this and what comes thereafter.

Line 225: "translation calls". You probably mean predicted translated ORFs.

Lines 287-288: "This correlation.. indicating that RPKM-FP can be a good proxy of protein-producing, productive canonical translation". Note that producing and productive are redundant here. In relation to the conclusion Fig. 3C shows that there is a relationship between proteomics-based quantification and RPKM-FP (R=0.45) but for high RPKM values only. This means that in the other RPKM ranges it would not be a good proxy. I think this result is interesting and should be explained in more detail.

Line 347: "However,..". I am not sure however is what is intended here.

Line 397: "significantly raised". Higher, maybe?

Line 477: "Whether uORFs act as cis-translational regulators,..". Something seems to be missing here.

Line 619: "short CDS.. do not show intermediate conservation". It may be more clear to say "are as poorly conserved as uORFs".

Line 663: "Translation levels appear more reproducible, yet highly correlated, with transcription". Does this mean more reproducible than transcription levels? This seems interesting, yet it is not reported in the results.

**Authors’ response to reviewers**

Reviewer 1: We thank this reviewer for the positive comments, and for sparing us from applying further pipelines. In Drosophila, unfortunately, the distribution of FPs sizes can be quite wide (Fig. S1A) and variable, and thus precludes the use of Ribo-Taper and Ribo-ORF (and other off-the-shelf pipelines), which rely on calculating P-sites and A-sites (we tried). Also, we feel more confident using simple statistic tools to define translated ORFs (such as the cumulative binomial probability) than other people´s functions (ORF-score, etc.), although we note that Ribo-Taper seems to use a reverse cumulative function (or tail function) to define ORFs with framing, which is essentially what we are using. Note nonetheless that we use common tools such as HISAT2, Bowtie, Bedtools, Samtools, and RiboSeqR. The methods section on detection of framing (page 23) has been corrected and expanded to clarify how we implement a binomial cumulative probability calculation. We have also clarified our pipeline in Fig. 2A.

Reviewer 2: We thank this reviewer for the informed and constructive comments, which have improved the paper.
Major comment 1: ´this (binomial) test (as many others) is going to be less sensitive for small ORFs than long ORFs´. We have considered this issue of sample size many times. First, we have found that all other methods we tried (see above) are also N-dependent (read number), and thus ultimately, size-dependent (ORF length). This is an issue of statistics (the larger the sample, the higher the confidence) and there is not much we can do about it. As the reviewer correctly points out, a 80, 20, 20 distribution of reads across frames for a given ORF will give more confidence (lower p-value) than a tenfold decrease in read number keeping the same proportions (8,2,2); but we have come to the conclusion that it is right for this to happen. At the gut level, and based on experience (forget statistics for a minute) a result based on 120 observations feels more solid and reproducible than one based on 12, isn´t it? However, we found that the binomial distribution can still give you reasonable confidence with very low samples, or number of reads, making it ideal for lowly translated ORFs like smORFs (unlike other methods, see above response to Reviewer 1). Hopefully, colleagues using our data will look at the tables with p-values that we provide, and conclude that they should rather follow-on ORF1 with p=0.001, and that they should corroborate experimentally the translation of ORF2 with p=0.099 before following it on as well. Certainly, we will.
We provide in the manuscript further evidence that smORFs do not seem discriminated against by the binomial test, and that the differences between translated and Ribo-bound are bona-fide:
1.1) We replaced Fig. 4F (now in Fig. S2D) with the old Fig S3C showing the overlapping distribution of RPKM values in ribosome-bound and translated uORFs, but adding the median size of the two uORF populations, Ribo-bound and Translated. They are very similar, indicating that neither ORF size, nor RPKM (normalised amount of signal) fully separates the two populations. (Note also that RPKM actually ´increases´ the ´normalised signal´ of features <1 kb, and this effect benefits more the smaller ORFs). Here you have the absolute read comparison, showing that Ribo-bound uORFs have similar numbers of reads, or even more, than translated:

1.2) We replaced Fig. 4E (now in Fig. S2C) with a genome-wide metagene framing for Ribo-bound uORFs (excluding translated uORFs), which shows un-framed signal quite similar to RNA-seq reads (with a frameshift that we discussed before, but had not showed). If the Ribo-bound uORF population were composed of actually translated uORFs whose low signal just hovers below the binomial filter, the metagenomics superposition of all their signals would show a framed profile; that is not the case (the metagene framing of translated uORFs is provided in Fig. S3C for comparison).

Major comment 2) ´what happens when there are several overlapping uORFs?´ The text in methods on “ORF selection and identification” (Lines 707-721) has been changed for clarity. uORFs fully overlapping (i.e. included) inside another uORF, and in the same frame, are discarded (GetORF can interpret internal AUGs as starts). Overlapping uORFs in different frames are preserved, and their RPKM and framing studied. This case is relevant to comment 1 above, and although we already discussed this possibility as a source of loss of framing (Line 552) we now mention that there are 302 Ribo-bound uORFs (out of 7,088, or 4.2%) which overlap translated uORFs. These overlapping uORFs could actually be totally inert, but appear as Ribo-bound due to the FPs that in reality might be generated by the translation of the overlapping uORF. However, they are still ‘ribosomal bound’ and as such, they have not been excluded from subsequent analyses.
Major point 3: ´The abstract states: …the main function of uORFs is to buffer canonical ORF translation. This seems an overstatement´. As we indicate in the discussion, the buffer hypothesis is also compatible with other observations, such as the higher TE of uORFs vs. main ORFs (Fig. 4B), and the frame-shifted signal observed in ribosome-bound-only uORFs (Fig. 3F). Nonetheless, these are compatible with other explanations, so the abstract has been amended as suggested.

Minor comments:
Line 134: amended as indicated.
Line 135: Text expanded and clarified.
Line 181-183: The tal ORFs are more precisely mapped, panel enlarged, and ORF size indicated. The text and legend now clarify that the FPs map to translated ORFs, as expected, and refers to Sup. Table 3A for further details.
Line 190: These sentences have been rewritten to clarify. Applying RPKMFP> 1 to both replicas selects signal that is reproducibly above background.
Line 223: This section, and methods about footprint analysis, have been expanded to clarify that in reality, more FPs (of 26 to 36 nt) than those used to measure framing (of 32nt only) map to translated ORFs. (see response to reviewer 1). This is also relevant to major point 1 above. We are discarding ORFs with low signal. We believe that the best definition at the moment for low-signal ORFs is Ribo-bound. Some could be shown to be productively translated into peptides in further studies, but this will complete, not rectify, our current classification (since Ribo-bound is a requisite to be translated).
Line 225: We meant translation of a given ORF in a given developmental stage. We have changed “calls” to “ORF translation events in a given stage”.
Lines 287-288: We have also studied this for a long time. The reviewer is correct, and the text has been amended. “protein producing, productive canonical translation” actually means, in plain English, “translation of canonical ORFs at moderate to high levels”. We have also modified Fig. 3C to show that, if the data is binned for RPKM intervals, the correlation between Poly-Ribo-Seq and quantitative MS data breaks down below RPKM=1 (log2=2) and above 1,024 (log2=10).
Line 347: This section has been expanded to clarify that Fig. 4A is a qualitative analysis (translated vs. not translated) and includes, by necessity, the effects of both transcriptional and translation regulation (which is of interest from a developmental gene expression perspective that is interested in what proteins are expressed when, for whatever reasons); whereas the subsequent studies based on TE analyse exclusively the effect of translational regulation.
Line 397: Clarified that it is “significantly raised according to Z-ratios” as the previous analyses of Figs. 4 and 5. ´raised´ and ´lowered´ indicate the tendency from a previous temporal point, whereas ´higher´ does not have temporal information.
Line 477: English corrected.
Line 619: Corrected as suggested.
Line 663: the reviewer´s interpretation is as intended. The phrase has been re-ordered to clarify. The higher reproducibility of translation is discussed in the section “Comparison among genomic data” and in Figs. 1C and 3A and Sup. Table 2, and references therein. Since this section is the conclusions, we did not include figure calls or references.
Apart from the changes discussed above we have also improved the manuscript in a number of other ways, such as typos, improved language for clarity, and improving the coherence of numeric results between Text, Figures, and Tables.
